# Evolutionary Process of Promoting Construction Safety Education to Avoid Construction Safety Accidents in China

**DOI:** 10.3390/ijerph181910392

**Published:** 2021-10-02

**Authors:** Feng Guo, Junwu Wang, Denghui Liu, Yinghui Song

**Affiliations:** 1 School of Civil Engineering and Architecture, Wuhan University of Technology, Wuhan 430070, China; guofeng777@whut.edu.cn (F.G.); 267544@whut.edu.cn (J.W.); songyinghui@whut.edu.cn (Y.S.); 2 Sanya Science and Education Innovation Park, Wuhan University of Technology, Sanya 572025, China; 3 China Construction First Group Corporation Limited, Beijing 100161, China

**Keywords:** construction safety education, trilateral evolutionary game, simulation analysis, reward and punishment mechanism

## Abstract

Construction safety is related to the life and health of construction workers and has always been a hot issue of concern for government and construction units. The government can use “construction safety education” to reduce the probability of safety accidents in the construction process and avoid the loss of life and property of construction workers. To encourage construction units to provide safety education for construction workers before construction starts and promote construction workers to actively participate in safety education. In this paper, a tripartite evolutionary game model of government–construction units–construction workers is established, and the factors affecting each party’s behavior strategy are comprehensively analyzed. Firstly, evolutionary game theory is used to investigate the influence of different behavior strategies among government, construction units, and construction workers on the behavior strategies of the other two parties. Secondly, according to the events in different situations, the influence of critical factors on the evolution process of the model is analyzed. On this basis, combined with the construction experience and construction data of actual construction projects, the established model and preliminary conclusions are verified. Finally, a sensitivity analysis of all parameters is carried out. The results show that: (1) The government’s enhancement of reward and punishment is conducive to promoting the choice of "providing safety education" for construction orders and the choice of “actively participating in safety education” for construction workers, but the excessive reward will lead to the government’s unwillingness of participation; (2) The reasonable reward and punishment mechanism set by the government must meet the condition that the sum of rewards and punishments for all parties is more significant than their speculative gains, to ensure the construction safety under the evolutionary stability; (3) Increasing welfare subsidies for construction workers who choose to participate in safety education actively is an effective way to avoid unwilling participation of construction workers.

## 1. Introduction

With the development of urbanization and population growth, China’s demand for construction is also increasing. Since 2013, China has built more than 4 billion square meters of buildings every year, and this construction speed is expected to continue in the next few decades, with an increase of 33 billion square meters by 2040 [1]. Such a huge demand for construction will bring many job opportunities, leading to more people engaging in the construction industry. However, developers usually tend to quickly complete the project to maintain the rapid pace of economic and social development [2]. However, rapid completion aside, developers have to consider the probability of construction safety accidents. Compared with other industries, the accident rate of the construction industry is very high [3,4,5]. The construction industry has only about 7% share of the global labour force, but it causes 30–40% of the work deaths [6]. Therefore, China has issued a series of measures on safe and civilized construction to ensure on-site construction safety, among which strengthening the safety education of construction workers is an important means [7].

Why should our country concern about the issue of safety education for construction workers? First of all, in China, the United States, Singapore and some other countries, top management and labour services in the construction industry are separated. Construction units usually subcontract their contracted labour operations to subcontractors with corresponding qualifications. Then, these labour subcontracting units organize the labor force to carry out construction and provide technical guidance [8]. Usually, these workers choose to join the construction company simply for employment purposes. However, they are generally hired as temporary workers, thus making the construction unit unwilling to waste time and money providing them with safety education. To improve this situation, the government has to consider possible measures to enhance the motivations of the construction units and workers to carry out and participate in construction safety education, which can be understood as on-the-job training and aims to provide construction workers with safety knowledge and theory.

Second, it has been widely reported that construction safety education is critical for improving the safety performance of the construction industry [9]. In the United States, about 1/4 of fatal work-related injuries occur in the construction industry every year. Some scholars believe that this is due to the lack of safety education [10]. India has the highest probability of safety accidents among construction workers in the world. According to the international labour organization statistics, 165 workers are injured at work for every 1000 workers. The main reason behind this is that most workers have are rarely provided safety education. The above reviews demonstrate the importance of construction safety education and vocational skill training as they have been proven effective in reducing accidents.

Finally, in China, apart from the widespread issue of temporary employment in the workforce described in the second paragraph, the other fact is most of the construction units are at a small scale with insufficient capability on providing safety education. In addition, the competition from their peers and complicated contracting and subcontracting relationship increases the difficulty of small enterprises in implementing health and safety measures [11]. Moreover, considering the gradual decline of China’s demographic dividend [12], the positive transformation of economic structure and the upgrading of related industries, labor-intensive industries requiring high-quality labor are often ignored [13,14]. These factors make it very important to improve the quality of construction workers. Therefore, providing relevant safety education for construction workers is not only to reduce the occurrence of safety accidents on the construction site but also to promote the further development of China’s construction industry.

To date, lots of work has been done in the literature to address these issues. However, the author found that most of the current studies mainly focus on the factors affecting construction safety [15], the impact of safety education on construction safety [16], and the selection of safety education suitable for construction workers [17,18]. Although the types of safety education provided by the construction units have important research value, according to the author’s field research, the effect of safety education provided by most construction units is very poor, not because they do not understand the correct and effective safety education methods, but owing to the fact that they do not want to provide high-quality construction safety education. Therefore, this paper mainly studies the following two problems. First, to determine the measures helping further promote the development of construction safety education in China. Second, to explore the impact of this development on the strategy choice of participants. In the process of promoting the development of construction safety education, the construction unit is considered to be the most important management object of the government and has direct responsibility for safety education, and the construction workers are the main participants in construction safety education. Therefore, different from other studies, this paper aims at reducing the probability of safety accidents. The paper studies the effective measures that all the units involved in the construction should take to promote the construction units to provide safety education and promote the active participation of construction workers in safety education. This is of great significance to reduce the accident rate and avoid the loss of life and property of construction workers.

The main structure of this paper is as follows. The second chapter is a review and summary of the literature. The third chapter is about the introduction and establishment of a game model of government–construction unit–construction workers. In the fourth chapter, the evolutionary stability strategy (ESS) of the model is analyzed. In the fifth chapter, we combine the experience of an actual project and the data of the relevant construction contract to carry out a numerical simulation analysis and discuss the influence of some relevant parameters on the evolution results of the game model. In the sixth chapter, the authors further discuss and analyze the established model with the assist of previously reported model analysis and numerical simulation results, which help build a foundation for the subsequent relevant policy development. Finally, the last chapter is a summary of the work, some constructive implications for the government, and limitations are given. The research is of guiding significance to promote safety construction, formulating incentive policies for the government and enhancing the industry’s competitiveness to build stakeholders.

## 2. Literature Review

### 2.1. Construction Safety Education

In China, the provision of safety education and training is required by law. This law is included in the “Regulations on the administration of construction project safety” and “law of the people’s Republic of China on work safety” of all provinces and cities in China. The regulations require that safety education must be organized before the commencement of the project, or safety education and production training must be conducted once a year. China is not the only country to enforce safety education. In the UK, developers also have to ensure that construction workers are trained for the task. Hong Kong and Singapore have similar compulsory safety education systems [19], and the United States also has laws related to workers’ safety education [20]. All of these illustrate the importance that the nations attach to construction safety education.

In the last century, some scholars found that safety education for construction workers is one of the best measures to improve the construction industry’s safety [21,22,23]. Following them, there are also relevant conclusions to confirm this. Wilkin found that health and safety education programs have improved the health of construction workers. The trainees’ response was more favorable when meaningful learning theories were integrated into these programs [24]. Oswald et al. found that safety education was a major factor in improving the safety atmosphere, safety concept and safety behaviour of construction projects [25,26]. In addition, many scholars, with an understanding of the importance of construction safety education, have also begun further research the use of emerging technologies to enhance construction safety education. Many researchers have explored how to use network-based tools, such as video and virtual reality, to strengthen construction safety education [27,28,29,30].

Although safety education has been pointed out as an important measure to reduce the probability of safety accidents, some scholars found that compared with many low-risk industries such as catering, the construction industry has less investment in safety education [31]. In fact, as described above, many small construction units only provide simple or even no safety education, which may be caused by construction project duration constraints and cost pressure and other factors, which make any training become a burden for them [32].

In sum, although there is significant research on the importance of building safety education, many of them are based on interviews or managers’ views. They do not consider the direct or indirect effects of different strategies among individual participants on the other participants, nor do they take into account the various effects of the influencing factors on different participants. As the purpose of this paper is to determine reasonable and practical measures to promote the construction unit to actively provide safety education and encourage construction workers to participate in safety education actively. This paper considers using a suitable mathematical model to deal with these uncertainties and to clarify the relationship between the government’s willingness to participate, the construction units’ desire to provide safety education, and the construction workers’ willingness to participate in these training. Obviously, evolutionary game theory is a proper option and is therefore adopted in this work.

### 2.2. Application of Evolutionary Game Theory

Evolutionary game theory is a theoretical method to determine how bounded rational participants make decisions under the background of incomplete information [33]. This method emphasizes the dynamic balance of the whole system. At present, many scholars are now using evolutionary game theory to solve problems in various fields, including economics [34], computer science [35], and management [36]. In architecture, many scholars use evolutionary game theory to solve related problems. Chen used evolutionary game theory to provide insights for promoting green building policy in China and provided suggestions for building stakeholders to maintain market competitiveness [37]. Li uses evolutionary game theory to provide method suggestions for further promoting prefabricated buildings in China [38]. Evolutionary game theory can be used to analyze the influence of each parameter in the game model on players’ decision-making behaviour and reveal the evolutionary path of their strategic choice. In addition, different from the classical game theory, evolutionary game players constantly observe and imitate each other in the process of interaction so as to optimize the strategy [39]. Therefore, the authors believe that evolutionary game theory is an excellent method to clarify the relationship between the three players. The application of evolutionary game theory determines the strategic stability of the government, construction units, and construction workers, qualitatively analyzes the factors affecting the change of the tripartite strategy, and quantitatively analyze how to change the size of each influencing factor, so as to achieve the ideal strategic stability.

In the literature, most papers using evolutionary game theory only focus on the two-party game, such as government and developers [35] or developers and consumers [36], without introducing the influence of the third party. In addition, the conclusion of the existing evolutionary game theory papers on construction safety education is that if the government does not participate, construction units usually do not provide safety education [39]. However, these studies do not consider that even if the government does not participate in safety education, construction units can also realize the high fines from the government in case of safety accidents. Therefore, for construction units, providing safety education is also an effective measure to avoid such fines. Therefore, it is of great significance to take the basic concept that safety education can reduce the incidence of safety accidents into consideration to understand all parties’ strategy choices.

Based on previous research and the new perspective of China’s high construction safety accident fines, the primary research significance of this paper is to improve China’s construction safety education level through the method of the evolutionary game to avoid the occurrence of safety accidents on the construction sites. The penalty system for safety accidents developed in China is a market governance mechanism that effectively complements government regulation. The evolutionary game model can verify this in the following aspects: (1) Expanding the effectiveness of the government’s current law, reflecting the diversity of regulatory approaches and regulatory effectiveness. (2) Forcing construction units to fulfil their safety responsibilities and enhancing the self-safety awareness of all parties involved. (3) Forming a safe environment for good production and promoting the healthy development of the construction industry. In summary, it is feasible to establish a tripartite evolutionary game model involving the government–construction unit–construction workers to clarify the relationship between the strategic choices of these three parties in promoting construction safety education.

## 3. Evolutionary Game Model

The game players in this study are the government, construction units and construction workers, and they are bound, rational decision-makers. The government bears the social responsibility of creating a better living environment for citizens, so any safety accidents in construction projects are beyond its tolerance. However, as the direct stakeholders of construction projects, construction units and construction workers always seek the maximum profit for themselves in various situations. This leads to the emergence of the rush period, no safety education and other phenomena. The strategic choice of government, construction unit and construction workers in the construction process, will affect the behaviour of the other two parties. To make the model more practical, we put forward the following six hypotheses.

### 3.1. Model Hypothesises

**Hypothesis** **1:** *The government, construction unit and construction workers take strategies independently and change strategies dynamically. The only criterion for making a choice is to maximize the profit that can be achieved. However, their focus is different. Construction units and workers focus on maximizing economic profits. At the same time, the government puts social benefits in the first place because protecting the safety of citizens is an important part of its responsibilities*.

**Hypothesis** **2:** *All three parties have two strategies. For the government, one strategy is to participate in construction safety education, and the other is not to participate in construction safety education. Construction units can choose to provide safety education or not. Construction workers can choose to participate in safety education actively or passively. If the construction units choose not to provide safety education, construction workers then have no choice. The promising strategy for these three parties is participation, provision, active participation, and the negative strategy is non-participation, non-provision, negative participation. Active participation means that workers will attend all safety education provided by the construction company. In contrast, negative participation implies that workers will avoid safety education and take up other part-time jobs*.

**Hypothesis** **3:** *The initial profit of the government comes from taxes, and the tax coefficient is defined as K. If the government chooses to participate in safety education, the construction units that choose to provide safety education and have no safety accidents will be rewarded with A, and the construction units that choose not to provide safety education will be fined with P_1_. If the government chooses not to participate in education, the construction units can determine strategies independently. When there is no safety accident in the construction process of the construction enterprise, it will bring enormous social benefits, represented by SB, to the government (for example, improving citizen satisfaction and sustainable development). When there is a safety accident, the construction unit will be fined P_2_ by the government, and the construction unit will deduct the bonus of construction workers, and the amount is P_3_. In the long run, social benefits will be greater than the cost of incentives (SB > A)*.

**Hypothesis** **4:** *The initial profit of the construction company is defined as M. if the construction company chooses to provide safety education, then it needs to pay an extra fee for this choice, which is denoted as T. At the same time, providing safety education can reduce the probability of safety accidents in the construction process. If the construction unit chooses not to provide safety education, it can obtain extra benefits A_1_*.

**Hypothesis** **5:** *If the construction unit chooses not to provide safety education, then the probability of safety accidents is α_1_ Otherwise, the probability of a safety accident is α_2i_ (when i = 1, construction workers choose passive participation, when i = 2, construction workers choose active participation)*.

**Hypothesis** **6:** *The initial profit of construction workers is defined as N. If the construction unit chooses to provide safety education and the construction workers choose to participate actively, they will get a long-term profit R, such as theoretical knowledge. If the government chooses to participate in safety education, the profit of construction workers is r (r > R), including additional wage subsidies. If the construction workers choose passive participation, they can take part-time jobs to obtain benefits, but they may face fines from the construction units, which is CP. If the construction unit chooses not to provide safety education, the construction workers will take part-time jobs and obtain benefits I*.

### 3.2. Model Establishment

**Figure 1:**
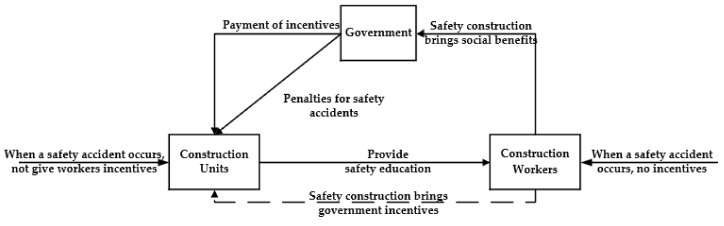
Tripartite Relationship Diagram of Government–Construction Unit–Construction Workers.

The tripartite relationship diagram is obtained based on the above hypotheses and the project’s actual situation, as shown in Figure 1, (the solid implies a direct influence and dashed line implies an indirect influence).

The construction units and construction workers have two strategies to respond to the government, which leads to six scenarios. Particularly, when the construction enterprise chooses not to provide safety education, the construction workers have no choice).

(1) When the government chooses to participate, construction units choose not to provide, and construction workers choose to participate actively. The probability of safety accidents in the construction process is $\alpha_{22}$. At this time, the interests of construction workers are $N+r\;-\alpha_{22}P_{3}$, the interests of construction units are $M\;-T\;-{\;\alpha }_{22}P_{2}+(1\;-\alpha_{22}{)A+\alpha }_{22}P_{3}$, and the government’s interests are $K\left[M\;-T\;-\alpha_{22}P_{2}+(1\;-{\;\alpha }_{22}{)A+\alpha }_{22}P_{3}\right]-(1\;-\alpha_{22}{)A+\alpha }_{22}P_{2\;}+(1\;-{\;\alpha }_{22})\mathrm{SB}$. Only the taxes paid by construction units are considered, and personal income tax has been deducted.

(2) When the government chooses to participate, construction units choose to provide, and construction workers participate negatively. The probability of safety accidents in the construction process is $\alpha_{21}$ At this time, the interests of construction workers are $N-\mathrm{CP}+I\;-{\;\alpha }_{21}P_{3}$, the interests of construction units are $M\;-\;T\;-{\;\alpha }_{21}P_{2\;}+(1\;-{\;\alpha }_{21}{)A+\alpha }_{21}P_{3}$, and the government’s interests are $K\left[M\;-\;T\;-{\;\alpha }_{21}P_{2\;}+(1\;-{\;\alpha }_{21}{)A+\alpha }_{21}P_{3}\right]-\;(1-{\;\alpha }_{21}{)A+\alpha }_{21}P_{2\;}+(1\;-{\;\alpha }_{21})\mathrm{SB}$.

(3) When the government chooses to participate, and the construction units choose not to provide, the construction workers have no choice at this time. The probability of safety accidents in the construction process is $\alpha_{22}$. At this time, the interests of construction workers are ${N+R\;-\;\alpha }_{22}P_{3}$, the interests of construction units are $M\;-\;T-{\;\alpha }_{22}P_{2}{+\alpha }_{22}P_{3}$, and the interests of the government are $K\left[M-T-{\;\alpha }_{22}P_{2}{+\alpha }_{22}P_{3}\right]{+\alpha }_{22}P_{2\;}+(1\;-{\;\alpha }_{22})\mathrm{SB}$

(4) When the government chooses not to participate, the construction units choose to provide, and the construction workers choose to participate actively. The probability of safety accidents in the construction process is $\alpha_{22}$. At this time, the interests of construction workers are $N+R\;-{\;\alpha }_{22}P_{3}$, the interests of construction units are ${M\;-\;T\;-\;\alpha }_{22}P_{2}{+\alpha }_{22}P_{3}$, and the interests of the government are $K\left[M\;-\;T-{\;\alpha }_{22}P_{2}{+\alpha }_{22}P_{3}\right]{+\alpha }_{22}P_{2}+(1-{\;\alpha }_{22})\mathrm{SB}$.

(5) When the government chooses not to participate, the construction units choose to provide, and the construction workers choose to participate passively. The probability of safety accidents in the construction process is $\alpha_{21}$. At this time, the interests of construction workers are $N\;-\;\mathrm{CP}+I\;-{\;\alpha }_{21}P_{3}$, the interests of construction units are $M\;-T\;-{\;\alpha }_{21}P_{2}{+\alpha }_{21}P_{3}$. The interests of the government are $K\left[M\;-\;T\;-\alpha_{21}P_{2}{+\alpha }_{21}P_{3}\right]{+\alpha }_{21}P_{2}+(1\;-{\;\alpha }_{21})\mathrm{SB}$

(6) When the government chooses not to participate, and the units choose not to provide, the construction workers have no choice at this time. The probability of safety accidents in the construction process is $\alpha_{1}$. At this time, the interests of construction workers are $N+I\;-{\;\alpha }_{1}P_{3}$, the interests of construction units are $M\;-{\;\alpha }_{1}P_{2}{+\alpha }_{1}P_{3\;}{+A}_{1}$, and the interests of the government are $K\left[M-{\;\alpha }_{1}P_{2}{+\alpha }_{1}P_{3\;}{+A}_{1}\right]{+\alpha }_{1}P_{2\;}+(1\;-{\;\alpha }_{1})\mathrm{SB}$. See Table 1 for detailed notations and Table 2 and Table 3 for the payoff matrix.

Based on the income matrix in Table 2 and Table 3, the probabilities are defined as follows. The probability that the construction workers actively participate in safety education is x, and the probability of passive participation is (1—x). The probability that the construction unit chooses to provide the safety education is y, and the probability of not providing safety education is (1—y). The probability that the government chooses to participate in safety education is z, and the probability of not participating is (1—z). The expected average income of the construction workers is W_1_. When the construction workers choose to take the safety education actively, the income is W_11_, otherwise, the income is W_12_.

(1) $$W_{11}=\mathrm{zy}(r\;-\;R)+y\left[R-I+P_{3}{(\alpha }_{1}-{\;\alpha }_{22})\right]+N+I\;-\alpha_{1}P_{3}$$

(2) $$W_{12}=y\left[P_{3}{(\alpha }_{1}-{\;\alpha }_{21})\;-\;\mathrm{CP}\right]+N+I\;-{\;\alpha }_{1}P_{3}$$

(3) $$W_{1}={\mathrm{xW}}_{11}+(1\;-{x)W}_{12}=W_{12}+x[\mathrm{zy}(r\;-\;R)+y[R\;-I+\mathrm{CP}+P_{3}{(\alpha }_{21}-{\;\alpha }_{22})]]$$

The expected average income of the construction unit is defined as W_2_. When the construction unit chooses to provide safety education, the income is W_21_; otherwise, the income is W_22_.

(4) $$W_{21}=\mathrm{xz}\left({A(\alpha }_{21}-{\;\alpha }_{22})\right)+\mathrm{zA}(1\;-{\;\alpha }_{21})+x[P_{2}(\alpha_{21}-{\;\alpha }_{22})+P_{3}(\alpha_{22}-{\;\alpha }_{21})]+M\;-\;T\;-{\;\alpha }_{21}P_{2}+\alpha_{21}P_{3}$$

(5) $$W_{22\;}=M\;-{\;\alpha }_{1}P_{2}+\alpha_{1}P_{3}+A_{1}-{\;\mathrm{zP}}_{1}\;$$

(6) $$W_{2}={\mathrm{yW}}_{21}+(1-{\;y)W}_{22}=W_{22}+y[\mathrm{xzA}(\alpha_{21}-{\;\alpha }_{22})+\mathrm{zA}(1\;-{\;\alpha }_{21})+x[P_{2}(\alpha_{21}-{\;\alpha }_{22})+P_{3}(\alpha_{22}-\alpha_{21})]\;-{\;T+(P}_{2}-{\;P}_{3}{)(\alpha }_{1}-{\;\alpha }_{21}{)+\mathrm{zP}}_{1}-{\;A}_{1}]$$

Similarly, the expected average revenue of the government is W_3_. When the government chooses to participate in safety education, the revenue is W_31_; otherwise, the revenue is W_32_.

(7) $$W_{31}=\mathrm{xy}\left[{K[(A+P}_{2}-{\;P}_{3})\left(\alpha_{21}-{\;\alpha }_{22}\right){]+(A+P}_{2}-\mathrm{SB})\left(\alpha_{22}-{\;\alpha }_{21}\right)\right]{+y[K[P}_{1}-{\;T+(P}_{2}-{\;P}_{3})\left(\alpha_{1}-{\;\alpha }_{21}\right)+A\left(1\;-{\;\alpha }_{21}\right)-{\;A}_{1}]+\;A\left(\alpha_{21}-\;1\right){+(P}_{2}-\;\mathrm{SB})\left(\alpha_{21}-{\;\alpha }_{1}\right)-{\;P}_{1}]+K\left[M\;-{\;P}_{1}-{\;\alpha }_{1}P_{2}{+\alpha }_{1}P_{3}{+A}_{1}\right]{+P}_{1}{+\alpha }_{1}P_{2}+(1\;-{\;\alpha }_{1})\mathrm{SB}$$

(8) $$W_{32}{=\mathrm{xy}[K(P}_{2}-{\;P}_{3})\left(\alpha_{21}-{\;\alpha }_{22}\right){+(P}_{2}-\;\mathrm{SB})\left(\alpha_{22}-{\;\alpha }_{21}\right)]+y[K\left(-{T+(P}_{2}-{\;P}_{3})\left(\alpha_{1}-{\;\alpha }_{21}\right)-{\;A}_{1}\right){+(P}_{2}-\;\mathrm{SB})\left(\alpha_{21}-{\;\alpha }_{1}\right)]+\;K\left[M\;-{\;\alpha }_{1}P_{2}{+\alpha }_{1}P_{3}{+A}_{1}\right]{+\alpha }_{1}P_{2}+(1\;-{\;\alpha }_{1})\mathrm{SB}$$

(9) $$W_{3}{=\mathrm{zW}}_{31}+(1\;-{\;z)W}_{32}{=W}_{32}{+z[P}_{1}(1\;-\;K)+\mathrm{xy}(K\;-\;1)A\left(\alpha_{21}-\alpha_{22}\right)+y(K\;-{\;1)[P}_{1}+A\left(1\;-{\;\alpha }_{21}\right)]]$$

The critical point of the evolutionary game is the dynamic change of strategy proportion [40]. The replicated dynamic equation is the most commonly used decision-making mechanism in the evolutionary game model, which was first proposed by Taylor and later developed and supplemented by Smith [41,42,43]. The basic form of the equation is $\dot{\dot{X_{i}}{=X}_{i}{(F}_{i}\left(x\right)-{\;\bar{F}}_{i}\left(x)\right)}$. In this model, $X_{i}$ is the frequency of making choices, $F_{i}\left(x\right)$ denotes the return in strategy i,$\;{\;\bar{F}}_{i}\left(x\right)$ represents the average return in the X state. The stable state and possible equilibrium point of the model can be determined by this method, which has been seen in many fields [44].

In the replicated dynamic equation, the parameter t is defined as time, and dx/dt is defined as the rate of change in the proportion of construction workers’ participation in safety education with time. According to Formulas (2) and (3), the replicated dynamic equation for determining the proportion of construction workers who actively participate in safety education can be expressed as follows.

(10) $$F(x)=\frac{\mathrm{dx}}{\mathrm{dt}}{=x(W}_{11}-{\;W}_{1})=(1-x)x[\mathrm{zy}(r\;-\;R)+y[R\;-{\;I+\mathrm{CP}+P}_{3}{(\alpha }_{21}-{\;\alpha }_{22})]]$$

The first derivative of x is:

(11) $$\frac{d(F(x))}{\mathrm{dx}}=(1\;-2x)[\mathrm{zy}(r\;-\;R)+y[R\;-{\;I+\mathrm{CP}+P}_{3}{(\alpha }_{21}-{\;\alpha }_{22})]]$$

Define $H\left(y,z\right)$ as:

(12) $$H\left(y,z\right)=\mathrm{zy}(r\;-\;R)+y[R\;-{\;I+\mathrm{CP}+P}_{3}{(\alpha }_{21}-{\;\alpha }_{22})]$$

According to the stability principle of relevant differential equations, the stable state of the probability that the construction workers choose to participate in safety education actively requires: F(x)=0, and $\frac{d(F(x))}{\mathrm{dx}}\;<\;0$. The partial derivative of z in H(y,z) can be obtained as $\frac{\partial H}{\partial z}=y(r\;-\;R)$, y(r −R) ≥ 0, i.e., H (y,z) is an increasing function of z. Therefore, when ${z=z}^{*}=(-1)\frac{[R\;-{\;I+\mathrm{CP}+P}_{3}{(\alpha }_{21}-{\;\alpha }_{22})]}{R^{’}-\;R}$, $H\left(y,z\right)=0$, that is $\frac{d(F(x))}{\mathrm{dx}}=0$, F(x)=0. At this point, all x are in an evolutionarily stable state. When ${z\;<\;z}^{*}$, $H\left(y,z\right)\;<\;0$, and x = 0, $\frac{d(F(x))}{\mathrm{dx}}\;<\;0$, indicating the evolutionary strategy of construction workers is stable at x = 0. Otherwise, when ${z\;>\;z}^{*}$, x = 1 is the evolutionary stable strategy. Therefore, the smaller z* is, the more likely z is to be greater than z*, and the more likely construction workers are to choose to participate in safety education actively. Therefore, the probability of construction workers actively participate in safety education is positively related to the income of participation (R, r), the penalty when they do not participate (CP), the penalty when safety accidents occur(P_3_), the probability of safety accidents when they do not participate in safety education (α_21_), and is negatively related to the part-time income (I) and the probability of safety accidents after participating in safety education (α_22_).

dy/dt is defined as the change rate of the proportion of safety education provided by construction units over time. According to Equations (5) and (6), the replicated dynamic equation for determining the proportion of safety education provided by construction units can be expressed as follows.

(13) $$F(y)=\frac{\mathrm{dy}}{\mathrm{dt}}{=y(W}_{21}-{\;W}_{2})=(1\;-{\;y)y[\mathrm{xzA}(\alpha }_{21}-{\;\alpha }_{22})+\mathrm{zA}(1\;-{\;\alpha }_{21}{)+x[(P}_{2}-{\;P}_{3}{)(\alpha }_{21}-\alpha_{22})]\;-{\;T+(P}_{2}-{\;P}_{3}{)(\alpha }_{1}-{\;\alpha }_{21}{)+\mathrm{zP}}_{1}-{\;A}_{1}]$$

The first derivative of y is:

(14) $$\frac{d(F(y))}{\mathrm{dy}}=(1\;-{\;2y)[\mathrm{xzA}(\alpha }_{21}-{\;\alpha }_{22})+\mathrm{zA}(1\;-{\;\alpha }_{21}{)+x[(P}_{2}-{\;P}_{3}{)(\alpha }_{21}-{\;\alpha }_{22})]\;-{\;T+(P}_{2}-{\;P}_{3}{)(\alpha }_{1}-{\;\alpha }_{21}{)+\mathrm{zP}}_{1}-{\;A}_{1}]$$

Define $G\left(x,z\right)$ as:

(15) $$G\left(x,z\right){=\mathrm{xzA}(\alpha }_{21}-{\;\alpha }_{22})+\mathrm{zA}(1\;-\alpha_{21}{)+x[(P}_{2}-{\;P}_{3}{)(\alpha }_{21}-{\;\alpha }_{22}{)]\;-\;T+(P}_{2}-{\;P}_{3}{)(\alpha }_{1}-{\;\alpha }_{21}{)+\mathrm{zP}}_{1}-{\;A}_{1}$$

The partial derivative of z in G(x,z) is $\frac{\partial G}{\partial z}{=\mathrm{xA}(\alpha }_{21}-{\;\alpha }_{22})+A(1\;-{\;\alpha }_{21}{)+P}_{1}$. Considering ${A(\alpha }_{21}-\alpha_{22})\;>\;0$, $\frac{\partial G}{\partial z}$ is an increasing function of x. When x = 0, $\frac{\partial G}{\partial z}=A(1\;-{\;\alpha }_{21}{)+P}_{1}\;>\;0$, indicating $G\left(x,z\right)$ is an increasing function of z. Hence, when ${z=z}^{*}=(-1)\frac{{x[(P}_{2}-{\;P}_{3}{)(\alpha }_{21}-{\;\alpha }_{22}{)]\;-\;T+(P}_{2}-{\;P}_{3}{)(\alpha }_{1}-\alpha_{21})\;-}{{\mathrm{xA}(\alpha }_{21}-{\;\alpha }_{22})+A(1\;-{\;\alpha }_{21}{)+P}_{1}}$, $G\left(x,z\right)=0$, i.e., $\frac{d(F(y))}{\mathrm{dy}}=0$, F(y)=0. At this point, all the y are in a stable state of evolution. When ${z\;<\;z}^{*}$, $G\left(x,z\right)\;<\;0$, at the same time, $\frac{d(F(y))}{\mathrm{dy}}\;<\;0$ when y = 0. Thus the evolutionary strategy of the construction unit is at a stable state when y = 0. Otherwise, the stable state occurs at x = 1 when ${z\;>\;z}^{*}.$ Accordingly, the smaller the z* is, the more likely z is to be greater than z*, and the more likely the construction unit is to choose to provide safety education. Therefore, the probability of safety education provided by the construction unit is positively related to the probability of construction workers actively participating in safety education (x), the reward given by the government (A), the fine imposed by the government for not carrying out safety education (P_1_), the fine in case of safety accident (P_2_), and the probability of safety accident in case of not providing safety education (α_1_), and it is negatively related to the income when the safety education is not provided (A_1_), the extra cost of safety education (T), the fine to the construction workers after the safety accident (P_3_), and the probability of safety accident after the safety education is provided ($\alpha_{2i}$).

Similarly, dz/dt is defined as the change rate of the proportion of government’s participation over time. According to Equations (8) and (9), the replicated dynamic equation for determining the proportion of the government’s choice of participation can be expressed as follows.

(16) $$F(z)=\frac{\mathrm{dz}}{\mathrm{dt}}{=z(W}_{31}-W_{3})=(1\;-{\;z)z[P}_{1}(1-\;K)+\mathrm{xy}(K\;-1)A\left(\alpha_{21}-{\;\alpha }_{22}\right)+y(K\;-{\;1)[P}_{1}+A\left(1\;-{\;\alpha }_{21}\right)]]$$

The first derivative of z is:

(17) $$\frac{d(F(z))}{\mathrm{dz}}=(1\;-{\;2z)[P}_{1}(1\;-K)+\mathrm{xy}(K\;-\;1)A\left(\alpha_{21}-{\;\alpha }_{22}\right)+y(K\;-{\;1)[P}_{1}+A\left(1\;-{\;\alpha }_{21}\right)]]$$

Define $U\left(x,y\right)$ as:

(18) $$U\left(x,y\right)=P_{1}(1\;-K)+\mathrm{xy}(K\;-\;1)A\left(\alpha_{21}-\alpha_{22}\right)+y(K\;-{\;1)[P}_{1}+A\left(1\;-{\;\alpha }_{21}\right)]$$

The partial derivative of y in U(x,y) is $\frac{\partial U}{\partial y}=x(K\;-\;1)A\left(\alpha_{21}-{\;\alpha }_{22}\right)+(K\;-{\;1)[P}_{1}+A\left(1-{\;\alpha }_{21}\right)]$. Considering $(K\;-\;1)A\left(\alpha_{21}-{\;\alpha }_{22}\right)\;<\;0$, $\frac{\partial U}{\partial y}$ is a decreasing function of x. When x = 0, $\frac{\partial U}{\partial y}=(K\;-{1)[P}_{1}+A\left(1\;-{\;\alpha }_{21}\right)]\;<\;0$, i.e., $\frac{\partial U}{\partial y}\;<\;0$, indicating $U\left(x,y\right)$ is a decreasing function of y. Hence, when ${y=y}^{*}=\frac{P_{1}}{\mathrm{xA}\left(\alpha_{21}-{\;\alpha }_{22}\right){+[P}_{1}+A\left(1\;-{\;\alpha }_{21}\right)]}$, $\frac{d(F(z))}{\mathrm{dz}}$ and F(z)=0. At this point, all the z are at a stable state of evolution. When ${y\;>\;y}^{*}$, $U\left(x,y\right)\;<\;0$, at the same time, $\frac{d(F(z))}{\mathrm{dz}}\;<\;0$ when z = 0. Thus the evolutionary strategy of the construction unit is in a stable state when z = 0. Otherwise, the stable state occurs at z = 1 when ${z\;<\;z}^{*}.$ Therefore, the larger the Y* is, the more likely y is to be less than y*, and the more likely the government is to choose participation. The probability of the government participating in safety education is positively related to the probability of safety accidents after providing safety education($\alpha_{2i}$), the penalty of the government for not providing safety education(P1), and negatively related to the probability of the construction workers actively participating in safety education(x), and the reward given by the government to the construction unit(A).

From the above, for each replicated dynamic equation, although the factors affecting each replicated dynamic equation have been analyzed, it is necessary to find the equilibrium point further and analyze the specific situation of different equilibrium points.

### 3.3. Model Solution

When the replicated dynamic equation is equal to 0, the model is stable and stops evolving [45], and the solution of the equation is the equilibrium point. According to Hirshleifer [46], in evolutionary game models, evolving the model from an arbitrarily small point to an asymptotically stable equilibrium point is called evolutionary stable strategy (ESS). The system remains stable if a sufficient proportion of players adopt the strategy of evolving to the same stable point to achieve the ESS. A stable point means that the model will reach stability and stop evolving when the strategies of all parties evolve to this point. The values of the stability points represent the strategies chosen by the players.

When F(x)=0, F(y)=0, F(z)=0, the solved equilibrium points are $E_{1}\left(0,0,0\right),$ $E_{2}\left(0,0,1\right),$ $E_{3}\left(0,1,1\right),$ $E_{4}\left(1,0,0\right),$ $E_{5}\left(1,1,0\right),$ ${\;E}_{6}\left(1,1,1\right),$ $E_{7}\left(1,0,1\right),$ $E_{8}\left(0,1,0\right),$ $E_{9}\left({0,y}_{9}{,z}_{9}\right),$ $E_{10}\left({1,y}_{10}{,z}_{10}\right),$ $E_{11}\left(x_{11}{,1,z}_{11}\right),$ where $y_{9}=\frac{P_{1}}{P_{1}+A\left(1\;-{\;\alpha }_{21}\right)},$ $z_{9}=(-1)\frac{{(P}_{2}-{\;P}_{3}{)(\alpha }_{1}-{\;\alpha }_{21})\;-\;T\;-A_{1}}{P_{1}+A\left(1\;-\alpha_{21}\right)},$ $y_{10}=\frac{P_{1}}{A(1-\alpha_{22}{)+P}_{1}},$ $z_{10}=(-1)\frac{{(P}_{2}-{\;P}_{3}{)(\alpha }_{1}-{\;\alpha }_{22})\;-\;T\;-{\;A}_{1}}{A(1\;-\alpha_{22}{)+P}_{1}}$, $x_{11}=\frac{\left(1\;-{\;\alpha }_{21}\right)}{\left(\alpha_{22}-{\;\alpha }_{21}\right)},$ $z_{11}=(-1)\frac{R\;-{I+\mathrm{CP}+P}_{3}{(\alpha }_{21}-{\;\alpha }_{22}))}{(R^{\prime }-\;R)}$.

According to the above model, it can be seen that $x,y,z\in \left[0,1\right]$, and whether the coordinates of E_9_~E_11_ are in this range needs further discussion. Considering $\frac{\left(1-\alpha_{21}\right)}{\left(\alpha_{22}-{\;\alpha }_{21}\right)}$ in E_11_ is obviously greater than 1, E_11_ is excluded. Generally, the stability condition can be derived by using the Jacobian matrix at the equilibrium point [47], which was proposed by Friedman. At present, the Jacobian matrix is as follows:


(19) $$J\;=\;\left[\begin{array}{ccc} \frac{\partial F(x)}{\partial x} & \frac{\partial F(x)}{\partial y} & \frac{\partial F(x)}{\partial z}\\ \frac{\partial F(y)}{\partial x} & \frac{\partial F(y)}{\partial y} & \frac{\partial F(y)}{\partial z}\\ \frac{\partial F(z)}{\partial x} & \frac{\partial F(z)}{\partial y} & \frac{\partial F(z)}{\partial z} \end{array}\right]\;=\;\left[\begin{array}{ccc} \lambda_{11} & \lambda_{12} & \lambda_{13}\\ \lambda_{21} & \lambda_{22} & \lambda_{23}\\ \lambda_{31} & \lambda_{32} & \lambda_{33} \end{array}\right]\;=\;\left[\begin{array}{ccc} \frac{\partial F(x)}{\partial x} & \left(1\;-x\right)x[z\tau_{1}\;+\;\tau_{2}] & \left(1\;-\;x\right)x[y\tau_{1}]\\ (1\;-\;y)y[\tau_{3}\;+\;z\tau_{4} & \frac{\partial F(y)}{\partial y} & (1\;-\;y)y[x\tau_{4}\;+\;\tau_{5}]\\ (1\;-\;z)z[y\tau_{6}] & (1\;-\;z)z[x\tau_{6}\;+\;\tau_{7}] & \frac{\partial F(z)}{\partial z} \end{array}\right]$$

For the convenience of demonstration, some functions are replaced by symbols. See Table 4 for details.

It can be noted that after substituting the above ten local equilibrium points into the Jacobian matrix, except $\lambda_{11}$, $\lambda_{22}$ and $\lambda_{33}$, the rest are 0, so whether the model can stabilize the equilibrium only needs to consider the values of these three. According to the existing research [48], scholars believe that when the eigenvalues of the Jacobian matrix are less than 0, the evolution reaches a stable state. Based on this, the values of ten equilibrium points are substituted into A1, A2 and A3, and the used values are shown in Table 5.

According to the value of each local equilibrium point in Table 5, one can further discuss the positive and negative of the value of each equilibrium point so as to obtain the stable relationship of the point. Based on hypotheses 1–6 and the definition of related symbols, the local stability of each equilibrium point can be obtained, as shown in Table 6.

For the convenience of the following discussion, the author defines $\tau_{8}=A(1\;-{\;\alpha }_{21}{)+P}_{1}{+(\alpha }_{1}-{\;\alpha }_{21}{)(P}_{2}-{\;P}_{3})\;-\;T-{\;A}_{1}$; $\tau_{9}=R\;-{\;I+\mathrm{CP}+P}_{3}{(\alpha }_{21}-{\;\alpha }_{22})$; $\tau_{10}={(P}_{2}-{\;P}_{3}{)(\alpha }_{1}-{\;\alpha }_{22})\;-\;T\;-{\;A}_{1}$; $\tau_{11}=A(1\;-{\;\alpha }_{22}{)+P}_{1}{+(P}_{2}-{\;P}_{3}{)(\alpha }_{1}-{\;\alpha }_{22})\;-T\;-{\;A}_{1}$; $\tau_{12}={\;\lambda }_{11}{(0,y}_{9}{,z}_{9})$; $\tau_{13}={\;\lambda }_{11}{(1,y}_{10}{,z}_{10})$.

## 4. Model Analysis

### 4.1. Model Analysis

(1) Situation 1 $\tau_{8}\;<\;0$ Passive participation, non-provision, participation. Situation 2 Active participation, non-provision, participation

When the construction company does not provide safety education, the government will choose to participate in safety education to change the behaviour strategy of the construction units and workers. In this case, the parameters that affect the construction units’ strategy selection are A, ${\;\alpha }_{22}{,P}_{1}{,\;P}_{2}{,\alpha }_{1}{,T,A}_{1}$. The corresponding evolutionary stability strategy is *arbitrary strategy, non-provision, participation*.

(2) Situation 3 $\tau_{9}\;>\;0$, ${\;\tau }_{10}\;>\;0$. Active participation, provision, nonparticipation. Situation 4 $\tau_{9}\;<\;0$, $\tau_{10}\;>\;0$. Passive participation, provision, nonparticipation.

When the construction units choose to provide safety education and the government departments do not participate in it, the model can be stable regardless of the choice of the construction workers. The reason is that if the construction units can actively choose to provide safety education, the government can avoid actively participating in safety education. In addition, the parameters that affect the construction units’ strategy selection as follows can be determined as $P_{2},P_{3}{,\;\alpha }_{1},\alpha_{22}{,\;T,\;A}_{1}$, and the parameters affecting construction workers’ strategic choice are ${R,\;\alpha }_{22},P_{3},\;{I,\;\mathrm{CP},\;\alpha }_{21},P_{3}$. In this case, the corresponding evolutionary stability strategy is the *arbitrary strategy, provision, nonparticipation*.

(3) Situation 5–6 $\lambda_{11}{(0,y}_{9}{,z}_{9})\;<\;0$, $\lambda_{11}{(1,y}_{10}{,z}_{10})\;>\;0$

Situations 5 and 6 are special cases with zero eigenvalues. When the other eigenvalues are negative, the equilibrium point is in a critical state, and its stability cannot be directly determined by the sign of eigenvalues.

From the above analysis, it can be concluded that when construction enterprises choose not to provide safety education, the government should take the initiative to participate to make the model stable. When construction enterprises actively provide safety education, the model can be stable even if the government refuses to participate. This is because the government hopes enterprises can actively strengthen the construction of safety education. Construction workers can actively participate in it to reduce the probability of safety accidents in the construction process and avoid the loss of life and property of construction workers. Although the positive and negative correlations between each parameter and the tripartite strategy have been discussed in Section 3.2, the model’s parameters need to be further summarized.

### 4.2. Parameter Discussion

In the model established in this paper, many basic parameters can cause the change of each party’s strategy. For example, whether τ_9_ above is greater than 0 may lead to situation 3 and situation 4. Therefore, it is necessary to analyze and discuss the parameters before the actual applications. See Table 7 for the specific impact of each parameter. (↑ represents active strategy *active participation, provision participation*, ↓ denotes passive strategy *passive participation non-provision nonparticipation)*.

(1) According to Table 7, except for parameters R, r, CP and I, all the other parameters can directly affect the strategic choice of construction units. Therefore, the government can adjust the strategic choice of construction units by adjusting the current reward and punishment system.

(2) It can be seen from Table 7 that the main parameters affecting the construction workers’ strategy selection are R, r, $\alpha_{21},$ $\alpha_{22}$, $P_{3}$, CP, I. Therefore, from the point of view that the construction unit wants to promote the construction workers to actively participate in safety education, adjusting the reward and punishment system related to the construction workers can improve the strategy choice of the construction workers.

(3) Also, one can see the main parameters affecting the choice of the government strategy is A, $\alpha_{21}$, ${\;\alpha }_{22}$,${\;P}_{1}$. Too much reward can hinder the government’s participation, and a high accident probability can make the government actively participate in it.

(4) In addition, the authors also found that only the parameters $\alpha_{21}$, $\alpha_{22}$ The accident probability when the construction units provide safety education affects the strategic choices of the three parties. However, the magnitude of each parameter may vary significantly. Hence, it is not reasonable to determine the importance of the parameters through the above table, and the proper determination method should refer to the actual numerical simulation and sensitivity analysis.

To sum up, the government’s reward and punishment system can change the behaviour strategy of the construction units and workers, and the reward and punishment system of the construction units can change the behaviour strategy of the construction workers. On the other hand, the strategy choices of the government and the construction units can also be affected by their reward and punishment systems. Therefore, in the process of strategy selection, the three parties should pay attention to the setting of the amount of rewards and punishments. Although the government can rely on high fines to make the construction enterprises provide safety education, such means are often counterproductive, and a more reasonable system of reward and punishment should be found. At the same time, other parameters should be set in a reasonable range to avoid too large or too small values.

## 5. Numerical Simulation

According to the hypothesis and analysis of the above model, the influence of parameters on the model results was analyzed in the above chapters. This section is about a numerical simulation used to test the results of the model. Nowadays, many scholars have used numerical simulation methods to conduct research [49].

However, some of the numerical values cannot be determined simply by surveying the literature, such as the probability of safety accidents, etc. Therefore, we conducted a questionnaire survey on the relevant employees of different types of projects, including Chengdu Metro Line 11 project, Wuhan Donghu deep tunnel project, Shuangyashan Chengxiang Construction Co., Ltd. The collected data was analyzed to determine the relevant parameters of the game model, and the influence of the parameters on the tripartite strategy was studied using MATLAB (2019a, MathWorks, Natick, MA, USA).

The evolutionary game model has a total of 14 relevant parameters. According to the regulations on the implementation of the enterprise income tax law of the people’s Republic of China, the general corporate income tax rate is 25% [50]. Tax calculation is a complex matter for a single company. To simplify the tax calculation, we assume that K remains a constant of 0.25. Other relevant parameters are set according to the production safety accident report, investigation and treatment regulations, and existing research papers. See Table 8 for details.

To verify the influence of different parameters discussed above on each party’s strategy choice, each parameter will be adjusted in the following to get the actual strategy choice diagram.

### 5.1. The Impact of R′ and R

**Figure 2:**
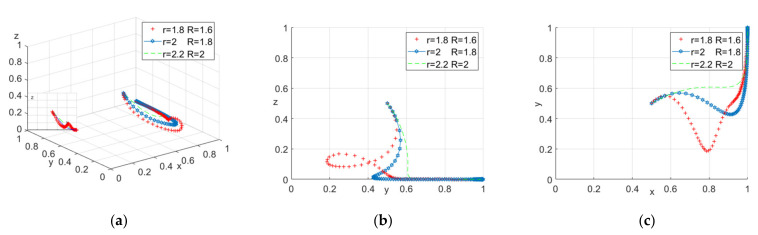
(**a**) Tripartite strategy; (**b**) Government–construction units; (**c**) Construction units–construction workers.

To analyze the influence of the benefits of safety education (r and R) on the process and result of the evolutionary game, we assigned r = 1.8, 2, 2.2, R = 1.6, 1.8, 2, respectively. The number of evolution times of the replicated dynamic equations was set as 50, and all the three parties evolved from 0. The simulation results are shown in Figure 2.

It can be seen from Figure 2 a that when the system evolves to a stable point with the increase of the profit of construction workers participating in safety education, the probability of construction workers’ active participation will increase, while the probability of government participation will decrease. This shows that under the current reward and punishment system, the construction workers will be willing to participate in safety education if the construction units can provide high-quality safety education and make the construction workers profitable. The government can also relax the supervision appropriately. Combining with Figure 2a–c, one can notice that the construction workers’ strategic choice fluctuates when their income is low. This may be because part-time income (I) can make them change their choice. In this case, the impact of part-time income is worth further research and analysis.

### 5.2. The Impact of I

**Figure 3:**
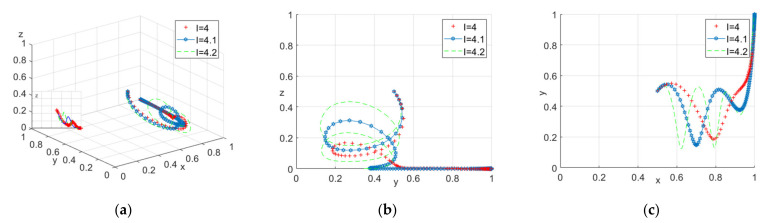
(**a**) Tripartite strategy; (**b**) Government–construction units; (**c**) Construction units–construction workers.

From Table 7, the part-time income (I) can affect the strategy choice of the construction workers. In this paper, we consider three different part-time incomes, i.e., I = 4, 4.1 and 4.2. See Figure 3 for details.

According to Figure 3, when the system evolves to a stable point, with the increase of part-time income, the choice of construction workers will fluctuate. Despite that, they will eventually actively participate in safety education. Therefore, the construction unit can consider increasing the punishment of not participating in safety education to deal with high part-time income.

### 5.3. The Impact of $A_{i}$, $P_{i}$ and CP

From Section 5.1 and Section 5.2, the income (R and r) and the change of part-time income (I) obtained by construction workers when they choose to participate in safety education will affect their choice. Considering that the relevant reward and punishment system affects the respective strategy selection of the three parties, it is of interest to further discuss and analyze the award and punishment system of the government and the construction unit.

(1) The impact of $A_{i}$

**Figure 4:**
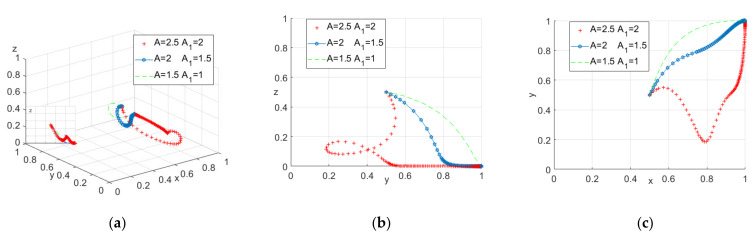
(**a**) Tripartite strategy; (**b**) Government–construction units; (**c**) Construction units–construction workers.

First, we only consider the impact of the reward A_i_ on the model results. When assigning A as 2.5, 2, 1.5, A_1_ as 2, 1.5, 1, respectively, the evolution of the model is shown in Figure 4.

As shown in Figure 4a, when the system evolves to a stable state, the construction units’ probability of providing safety education will increase as the incomes of the construction units (A and A1) decrease. This shows that even if the reward is reduced, the construction unit may still choose to provide safety education because the relevant punishment is too strong.

(2) The impact of $P_{i}$ and CP

**Figure 5:**
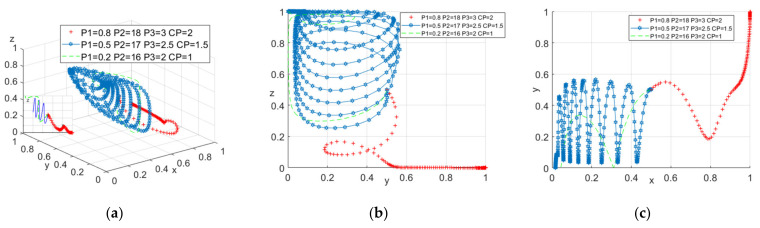
(**a**) Tripartite strategy; (**b**) Government–construction units; (**c**) Construction units–construction workers.

Secondly, in order to study whether the punishment in the initial parameters was set too large, we assigned CP as 2, 1.5, 1, P1 as 0.8, 0.5, 0.2, P2 as 18, 17, 16, P3 as 3, 2.5, 2, respectively. The evolution of the model is shown in Figure 5.

It can be seen from Figure 5a that when the system evolution is stable, with the reduction of the punishment, the probability of the construction units choosing not to provide safety education will increase, the likelihood of the construction workers’ negative participation will increase, and the probability of the government’s participation will increase. This further shows that if the construction units and workers do not choose a positive strategy, the government will come forward to control. Combining Figure 5a–c, it can also be concluded that the punishment of the government and the construction unit will make the government and the construction unit hesitate. In contrast, the construction workers will directly choose negative participation.

### 5.4. The Impact of α_1_ and α_2i_

**Figure 6:**
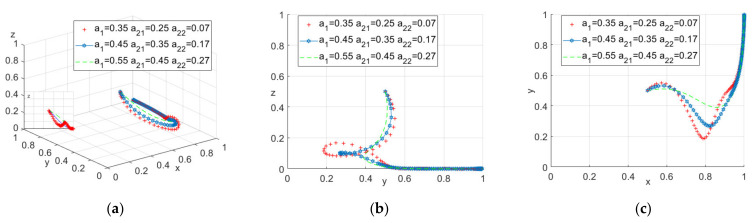
(**a**) Tripartite strategy; (**b**) Government–construction units; (**c**) Construction units–construction workers.

The initial parameter setting of accident probability in this paper was determined according to the actual construction process experience of the real construction project. However, because the situation of different areas and different construction projects may be different, and considering the randomness of the probability problem, the author intended to adjust the probability parameters of this model as follows. The $\alpha_{1}$ was assigned as 0.35, 0.45, 0.55, the $\alpha_{2}$ was assigned as 0.25, 0.35, 0.45, and the $\alpha_{22}$ was assigned as 0.07, 0.17, 0.27. See Figure 6 for the updated results.

It can be seen from Figure 6a that when the system evolves to a stable point, the impact of accident probability $\alpha_{1}$ and $\alpha_{2i}$ is basically consistent with the impact of construction workers’ participation profit R and r. With the increase of accident probability, construction workers’ probability of actively participating will increase, while the probability of government participation will decrease. Combining Figure 6a–c, it can also be seen that when the probability of accidents is low for construction units and workers, their strategy choices tend to fluctuate a lot.

To sum up, the author analyzes the model’s sensitivity in Section 5.1, Section 5.2, Section 5.3 and Section 5.4 from three aspects: the gain and loss of construction workers’ interests, the gain and loss of construction units’ interests, and the probability of accidents. Through comparative analysis, it is known that the government’s participation is beneficial for construction enterprises when they choose to provide safety education, and the government’s punishment measures are much more effective than the incentive measures. This conclusion can help the government to formulate relevant policies.

## 6. Discussion

### 6.1. Research Findings

In this paper, a tripartite evolutionary game model is constructed for theoretical analysis. Based on the analysis in this work, a total of 6 stability points are obtained and listed in Table 6, which are $E_{2}\left(0,0,1\right),\;E_{5}\left(1,1,0\right),\;E_{7}\left(1,0,1\right),\;E_{8}\left(0,1,0\right),\;E_{9}{(0,y}_{9}{,z}_{9}{),\;E}_{10}{(1,y}_{10}{,z}_{10})$. The stability point $E_{5}$ is the most idealized state from the perspective of government policymaking. In this state, the government does not need to be involved in safety education. The construction units will provide safety education, and the construction workers will actively participate in safety education spontaneously. After the accurate numerical simulation analysis in Chapter 5, the author found that the final stabilization point of this model stays at $E_{2}\left(0,0,1\right)$ and $E_{5}\left(1,1,0\right)$ in most cases. When the stability point is $E_{2}$, the government will participate in safety education. However, the construction units will not provide safety education, which is not ideal for government policymaking. Considering the research objective of this paper is mainly to promote the construction units to provide safety education and the construction workers to participate in safety education actively, the following discussion focuses on how to change the parameters so that the strategies of all the three parties can be changed to reach the ideal state. Some key findings are discussed as follows:

First, as discussed in Situation 3 of 4.1, the requirement for the model to evolve to an idealized state is $\tau_{9}\;>\;0$,${\;\tau }_{10}\;>\;0$. In this case, the following conditions should be met even without the involvement of the government. ${I+\mathrm{CP}+P}_{3}{(\alpha }_{21}-{\;\alpha }_{22}{)\;>\;0;\;(P}_{2}-P_{3}{)(\alpha }_{1}-{\;\alpha }_{21})\;-T\;-{\;A}_{1}\;>\;0$. Currently, the government has already set high fines $P_{2}$ for production safety accidents. It has strengthened the management of enterprises to some extent [51]. If the proportion of safety accidents rises each year, the government can adopt higher fines or even disqualify businesses to encourage enterprises to carry out safety production. Construction units can also impose fines on workers without motivation to participate in safety education [7]. However, punishment only and without considering reward may not lead to healthy sustainable development of construction units.

Second, besides setting punitive measures, additional bonuses and awards will enable construction units and workers to adopt positive strategies. In China, most of the construction projects are set up with incentives for safe and civilized construction, and construction workers can get specific bonuses in addition to individual honors to lay the foundation for future promotions [52]. As for construction units, the number of new construction projects conducting in China is enormous every year, causing it difficult to obtain additional rewards and awards from the government. Still, the government can provide certain tax breaks and other preferential measures to enterprises that meet the standards through assessment [53].

Third, the quality of construction safety education is a crucial factor affecting the attitudes of the construction units and workers. It can directly affect the probability of safety accidents. Although high-quality safety education is often accompanied by high additional expenditure T, it is insignificant compared to the loss of life and property and high fines in case of safety accidents. Therefore, in addition to monitoring the provision of safety education by construction companies, the government can also focus on the quality of safety education provided to promote the active participation of construction workers and reduce the probability of accidents.

Fourth, the parameters such as the income of construction workers going to part-time jobs I, the additional expenditure of providing quality safety education T, and the construction company’s income $A_{1}$ mostly depend on the overall market situation and cannot be simply determined by the three participants of the model. Nevertheless, the construction units can adjust their internal expenditure, such as the income of construction workers and bonuses, through specific market research to reduce the influence of the parameters I, T, $A_{1}$.

### 6.2. Our Research Versus Works in the Literature

This section describes the differences between our work and the studies existing in the literature on construction safety education. Scholars such as Pham [9] assessed the importance of construction safety education on safety performance in the construction industry. In this paper, based on identifying the importance of construction safety education, an evolutionary game model was used to investigate possible approaches to promote the provision of safety education by construction organizations and the active participation of construction workers. Loosemore et al. [54] found that most of these workers received mandatory site safety training through a survey of 228 construction workers. It was found that while such training led to a better understanding of safety risks and safety behaviors among construction workers, there was little change in their safety attitudes.

In contrast, this paper focuses more on enabling construction workers to actively participate in construction safety education by changing their attitudes and thus making them more concerned about safety issues. Sun et al. [7] studied the dynamic game relationship between construction units’ provision of safety education and construction workers’ willingness to participate in safety education. They concluded that government involvement is indispensable, and the construction unit will not provide such training if the government is not involved. However, they ignored the fact that construction units have to face significant penalties when safety accidents occur, even if the government is not involved. This paper considers the high government fine, making the model cover more affecting factors. In addition, Sun et al. [7] suggest that giving construction workers more financial subsidies and increasing wages will enhance their motivation, which is a common practice in China, but compared to receiving additional financial subsidies, construction workers also want to obtain better construction safety education to avoid safety accidents and improve their quality level. Hence, this paper argues that besides the subsidies, the quality level of the safety education provided should also be emphasized.

### 6.3. Theoretical Contributions

Promoting the development of construction safety education in China is a complex issue that requires the coordinated efforts of multiple parties. This paper identifies the conditions under which there are conflicting or consistent preferences among the government–construction unit–construction workers. Adopting the tripartite evolutionary game theory to promote construction safety education in China is first proposed in this work. This paper expands the application of evolutionary game theory, enriches the existing literature, and helps shift the focus of research from studying the importance of construction safety education to promoting construction safety education. This critical issue has been neglected previously. Additionally, this paper extends stakeholder research from two to three parties by introducing a new perspective on the high fines imposed by the Chinese government for construction safety accidents. Thus, the findings can complement existing research gaps by revealing the behavior of stakeholders in the process of promoting construction safety education.

## 7. Conclusions

The government’s decision-making can affect the construction units’ consideration of providing safety education and the construction workers’ enthusiasm to participate in safety education. In this paper, an evolutionary game theory model is used to study the optimal decision-making process in which the construction unit provides safety education and construction workers actively participate under the government reward and punishment mechanism. The paper also discusses the optimal strategies of construction workers, the construction units and the government in different situations. The main findings are as follows.

The reward and punishment mechanism of government and construction units directly or indirectly affects the strategy choice of construction workers, construction units and government.

The government’s reasonable reward and punishment mechanism must be in line with the sum of rewards and punishments for all parties and the conditions for their speculative income to ensure construction safety under evolutionary stability.

The government plays an important role in encouraging enterprises to provide safety education and construction workers to participate in the process of safety education actively. The government’s reward and punishment mechanism effectively standardizes the decision-making process of construction workers and the construction unit. Increasing punishment can help reduce negative behaviour, and increasing reward may make the government reluctant to participate in it.

The final stability strategy is sensitive to the parameters. The reasonable parameters should be determined according to the practical construction experience.

### Implications

From the above conclusions, the following management suggestions can be put forward. (1) Construction units should consider providing high-quality safety education to attract workers. (2) Construction units can also carry out relevant construction safety knowledge contests to mobilize the enthusiasm of construction workers. (3) A reasonable reward and punishment mechanism can encourage construction units to provide safety education and encourage construction workers to participate actively. If the government is not willing to spend too much on the reward, the alternative is to increase the punishment to ensure the safety of construction. (4) In addition, when the government chooses to be involved, it should supervise the construction units to provide safety education and pay attention to the quality of safety education.

With the evolutionary game model established in this paper, the government can determine a reasonable reward and punishment system by following the current mandatory standards and general construction contract regulations, which makes it possible for the government to promote the safety of construction projects.

However, the findings of this paper have limitations. For example, the study does not consider that some construction units may conceal the safety accidents on the construction site. The relevant parameters about the probability of accidents are not clear enough. Therefore, in future research, it is necessary to introduce the influence of accident concealment and supervision probability to build a dynamic game model.

## Figures and Tables

**Table 1:** Summary of notations.

Notations	Explain
R	Revenue from construction worker participation in safety education when the government is not involved.
r	Revenue from construction worker participation in safety education when the government is involved in safety education.
A	The bonus is given to the construction units that provide and have no safety accident when the government participates.
$A_{1}$	The income of construction units that choose not to participate.
M	Initial profit of construction units.
N	Initial profit of construction workers.
T	The extra expenditure when construction units choose to provide.
$\alpha_{1}$	Probability of construction safety accidents when the construction units refuse to provide construction safety education.
$\alpha_{2i}$	Probability of construction safety accidents when the construction unit provides construction safety education.
K	Coefficient of tax revenue.
SB	Social benefits are obtained by the government when there is no safety accident in the construction process.
CP	The penalty when the construction workers passively participate when the construction units choose to provide.
$P_{1}$	The penalty when the government chooses to participate and the construction units choose not to provide.
$P_{2}$	Fines faced by construction companies when construction safety accidents occur.
$P_{3}$	In case of a safety accident, the amount of bonus missing by construction workers.
I	Part-time income for construction workers.

**Table 2:** Payoff matrix for Government–Construction Unit–Construction Workers (government participates).

Construction Workers	Construction Units	
	Provide	Not Provide
Actively Participate	$N+r\;-{\;\alpha }_{22}P_{3}$; $M-\;T\;-{\;\alpha }_{22}P_{2\;}+(1-{\;\alpha }_{22}{)A+\alpha }_{22}P_{3}$; $K\left[M\;-T\;-\alpha_{22}P_{2\;}+(1-{\;\alpha }_{22}{)A+\alpha }_{22}P_{3}\right]-\;(1\;-{\;\alpha }_{22}{)A+\alpha }_{22}P_{2\;}+(1\;-{\;\alpha }_{22})\mathrm{SB}$	$N+I-{\;\alpha }_{1}P_{3}$; $M\;-P_{1}-{\;\alpha }_{1}P_{2}{+\alpha }_{1}P_{3}{+A}_{1}$; $K\left[M\;-P_{1\;}-{\;\alpha }_{1}P_{2}{+\alpha }_{1}P_{3}{+A}_{1}\right]{+P}_{1}{+\alpha }_{1}P_{2}+(1-\alpha_{1})\mathrm{SB}$
Passive Participate	$N\;-\mathrm{CP}+I\;-{\;\alpha }_{21}P_{3}$; $M-\;T-{\;\alpha }_{21}P_{2\;}+(1\;-{\;\alpha }_{21}{)A+\alpha }_{21}P_{3}$; $K\left[M\;-\;T\;-{\;\alpha }_{21}P_{2\;}+(1-{\;\alpha }_{21}{)A+\alpha }_{21}P_{3}\right]-\;(1\;-{\;\alpha }_{21}{)A+\alpha }_{21}P_{2\;}+(1-{\;\alpha }_{21})\mathrm{SB}$	

**Table 3:** Payoff matrix for Government–Construction Unit–Construction Workers (government not participate).

Construction Workers	Construction Units	
	Provide	Not Provide
Actively Participate	$N+R-\alpha_{22}P_{3}$; $M-\;T\;-\alpha_{22}P_{2}+\alpha_{22}P_{3}$; $K\left[M\;-\;T-\alpha_{22}P_{2}+\alpha_{22}P_{3}\right]+\alpha_{22}P_{2}+(1-\alpha_{22})\mathrm{SB}$	$N+I\;-\alpha_{1}P_{3}$; $M-{\;\alpha }_{1}P_{2}{+\alpha }_{1}P_{3}{+A}_{1}$; $K\left[M-\alpha_{1}P_{2}+\alpha_{1}P_{3}+A_{1}\right]+\alpha_{1}P_{2}+(1\;-\alpha_{1})\mathrm{SB}$
Passive Participate	$N\;-\;\mathrm{CP}+I\;-\alpha_{21}P_{3}$; $M-\;T\;-\alpha_{21}P_{2}{+\alpha }_{21}P_{3}$; $K\left[M\;-\;T-{\;\alpha }_{21}P_{2}{+\alpha }_{21}P_{3}\right]{+\alpha }_{21}P_{2\;}+(1-{\;\alpha }_{21})\mathrm{SB}$	

Attachment: The first formula in the cell represents the income of construction workers, the second formula represents the income of construction units, and the third formula represents the government income.

**Table 4:** Symbol description.

Symbol	Description
$\tau_{1}$	(r − R)
$\tau_{2}$	$R\;-{\;I+\mathrm{CP}+P}_{3}{(\alpha }_{21}-{\;\alpha }_{22})$
$\tau_{3}$	${(P}_{2}-{\;P}_{3}{)(\alpha }_{21}-\alpha_{22})$
$\tau_{4}$	${A(\alpha }_{21}-\alpha_{22})$
$\tau_{5}$	$A(1\;-{\;\alpha }_{21}{)+P}_{1}$
$\tau_{6}$	$(K\;-1)A\left(\alpha_{21}-\alpha_{22}\right)$
$\tau_{7}$	$(K\;-{1)[P}_{1}+A\left(1\;-\alpha_{21}\right)]$

**Table 5:** The value at each local equilibrium point.

Equilibrium	$\lambda_{11}$	$\lambda_{22}$	$\lambda_{33}$
$E_{1}\left(0,0,0\right)$	0	$-{T+(P}_{2}-{\;P}_{3}{)(\alpha }_{1}-{\;\alpha }_{21})\;-A_{1}$	$P_{1}(1\;-\;K)$
$E_{2}\left(0,0,1\right)$	0	$A(1\;-{\;\alpha }_{21}{)+P}_{1}-{\;T+(P}_{2}-P_{3}{)(\alpha }_{1}-{\;\alpha }_{21})\;-{\;A}_{1}$	$P_{1}(K\;-\;1)$
$E_{3}\left(0,1,1\right)$	$r\;-{\;I+\mathrm{CP}+P}_{3}{(\alpha }_{21}-\alpha_{22})$	$\left(-1)[A(1\;-{\;\alpha }_{21}{)+P}_{1}-{\;T+(P}_{2}-{\;P}_{3}{)(\alpha }_{1}-{\;\alpha }_{21})\;-{\;A}_{1}\right]$	$(1\;-K)A\left(1\;-{\;\alpha }_{21}\right)$
$E_{4}\left(1,0,0\right)$	0	${(P}_{2}-{\;P}_{3}{)(\alpha }_{1}-{\;\alpha }_{22})\;-\;T-{\;A}_{1}$	$P_{1}(1\;-\;K)$
$E_{5}\left(1,1,0\right)$	$(-1)[R\;-{\;I+\mathrm{CP}+P}_{3}{(\alpha }_{21}-{\;\alpha }_{22})]$	$\left(-{1)[(P}_{2}-{\;P}_{3}{)(\alpha }_{1}-{\;\alpha }_{22})\;-\;T\;-{\;A}_{1}\right]$	$(K\;-\;1)A\left(1-{\;\alpha }_{22}\right)$
$E_{6}\left(1,1,1\right)$	$(-{1)(R}^{’}-{\;I+\mathrm{CP}+P}_{3}{(\alpha }_{21}-{\;\alpha }_{22})]$	$\left(-1)[A(1\;-\alpha_{22}{)+P}_{1}-{\;T+(P}_{2}-{\;P}_{3}{)(\alpha }_{1}-{\;\alpha }_{22})\;-{\;A}_{1}\right]$	$(1\;-\;K)A\left(1-{\;\alpha }_{22}\right)$
$E_{7}\left(1,0,1\right)$	0	$A(1\;-{\;\alpha }_{22}{)+P}_{1}-{\;T+(P}_{2}-{\;P}_{3}{)(\alpha }_{1}-\alpha_{22})\;-{\;A}_{1}$	$P_{1}(K\;-1)$
$E_{8}\left(0,1,0\right)$	$R\;-{\;I+\mathrm{CP}+P}_{3}{(\alpha }_{21}-{\;\alpha }_{22})$	$\left(-1)[-{T+(P}_{2}-{\;P}_{3}{)(\alpha }_{1}-{\;\alpha }_{21})\;-{\;A}_{1}\right]$	$(K-\;1)A\left(1\;-{\;\alpha }_{21}\right)$
$E_{9}{(0,y}_{9}{,z}_{9})$	$\lambda_{11}{(0,y}_{9}{,z}_{9})$	0	0
$E_{10}{(1,y}_{10}{,z}_{10})$	$\left(-{1)\lambda }_{11}{(1,y}_{10}{,z}_{10}\right)$	0	0

**Table 6:** Local stability of equilibrium point.

Equilibrium	$\lambda_{11}$	$\lambda_{22}$	$\lambda_{33}$	State	Stable Condition
$E_{1}\left(0,0,0\right)$	0	−	+	Instability point	-
$E_{2}\left(0,0,1\right)$	0	+/−	−	Uncertain	$A(1\;-{\;\alpha }_{21}{)+P}_{1}-{\;T+(P}_{2}-P_{3}{)(\alpha }_{1}-\alpha_{21})\;-{\;A}_{1}\;<\;0$
$E_{3}\left(0,1,1\right)$	+/−	+/−	+	Instability point	−
$E_{4}\left(1,0,0\right)$	0	+/−	+	Instability point	−
$E_{5}\left(1,1,0\right)$	+/−	+/−	−	ESS (evolutionary stability strategy)	$R\;-{\;I+\mathrm{CP}+P}_{3}{(\alpha }_{21}-{\;\alpha }_{22})>0$; ${(P}_{2}-{\;P}_{3}{)(\alpha }_{1}-{\;\alpha }_{22})\;-\;T\;-{\;A}_{1}\;>\;0$
$E_{6}\left(1,1,1\right)$	−	+/−	+	Instability point	-
$E_{7}\left(1,0,1\right)$	0	+/−	−	Uncertain	$A(1\;-{\;\alpha }_{22}{)+P}_{1}{+(P}_{2}-{\;P}_{3}{)(\alpha }_{1}-{\;\alpha }_{22})-\;T\;-A_{1}\;<\;0$
$E_{8}\left(0,1,0\right)$	+/−	+/−	−	ESS(evolutionary stability strategy)	$R\;-{I+\mathrm{CP}+P}_{3}{(\alpha }_{21}-{\;\alpha }_{22})\;<\;0$; ${(P}_{2}-{\;P}_{3}{)(\alpha }_{1}-{\;\alpha }_{22})\;-\;T\;-{\;A}_{1}\;>\;0$
$E_{9}{(0,y}_{9}{,z}_{9})$	+/−	0	0	Uncertain	$\lambda_{11}{(0,y}_{9}{,z}_{9})\;<\;0$
$E_{10}{(1,y}_{10}{,z}_{10})$	+/−	0	0	Uncertain	$\lambda_{11}{(1,y}_{10}{,z}_{10})\;>\;0$

**Table 7:** Impacts of parameter change.

**Parameter**	Construction Workers	Construction Unit	Government
R↑	↑	-	-
r↑	↑	-	-
A↑	-	↑	↓
$A_{1}↑$	-	↓	-
T↑	-	↓	-
$\alpha_{1}↑$	-	↑	-
$\alpha_{21}↑$	↑	↓	↑
$\alpha_{22}↑$	↓	↓	↑
$P_{1}↑$	-	↑	↑
$P_{2}↑$	-	↑	-
$P_{3}↑$	↑	↓	-
CP↑	↑	-	-
I↑	↓	-	-

**Table 8:** Initial parameter setting.

Parameters	Values	Parameters	Values
K	0.25	$\alpha_{21}$	0.25
R	1.6	$\alpha_{22}$	0.07
r	1.8	$P_{1}$	0.8
A	2.5	$P_{2}$	18
$A_{1}$	2	$P_{3}$	3
T	2	CP	2
$\alpha_{1}$	0.35	I	4
